# Oral mucosal precancer and cancer: A helpful discriminating clinical tool

**DOI:** 10.4317/medoral.20155

**Published:** 2015-08-04

**Authors:** Crispian Scully, James J. Sciubba, Jose V. Bagan

**Affiliations:** 1 PhD, MD, DSc. Professor WHO Collaborating Center for Oral Health-General Health; and University College London, UK; 2 D.M.D., Ph.D.The M.J. Dance Head & Neck Center, and The Johns Hopkins School of Medicine, Baltimore, MD; 3 MD, DDS, PhD. Chairman of Stomatology and Head Department of Oral Surgery & Medicine, Valencia, Spain

## Abstract

The authors have collaborated with many colleagues in several countries in formulating a useful and practical clinical tool for evaluating oral mucosal findings on routine examination. Consideration of several factors including history, evolution of positive findings and clinical information allows placement of examination results into one of three categories which are graded by a color scheme along a spectrum of concerns (green to red, or no concern to serious concern). Afforded to the clinician is a straightforward grading system as a starting point for office end clinic use for all patients.

** Key words:**Oral, precancer, cancer, clinical tool.

The use of information technology in the area of clinical decision making is an increasing paradigm in the practice of medicine and dentistry. The technologies at hand within clinical decision support systems (CDSSs) have the potential to reduce clinical errors by incorporating systems for data analysis with that analysis used to measure and prevent adverse clinical outcomes, ultimately resulting in improved overall quality and efficiency in the delivery of care (1,2). Evidence-based medicine (EBM) - the practice of medicine based on the best available scientific evidence - can also improve clinical outcomes. The use of CDSSs to facilitate EBM therefore promises a substantial improvement in healthcare quality (3). EBM is based upon the scientific literature as the prime source of evidence, but should often be complemented by local, practice-based evidence for individual and site-specific clinical decision-making. However, lower levels of evidence and other sources such as Internet-based information should not be ignored (4). As stated recently in another context, “evidence-based recommendations are not a substitute for clinical judgment, and decisions about care must carefully consider and incorporate the clinical characteristics and circumstances of each individual patient” (5).

Early diagnosis remains the essential aim of the primary care clinician as the single-most important contribution to help avoid the need for major and possibly radical treatment and thus improving chances of cure and retention of function and quality of life.

We have therefore produced an “expert system” regarding evaluation of potentially malignant oral mucosal disease based upon considerable clinical experience on two continents, with advice from senior experienced colleagues. This was done in an effort to assist primary care clinicians in decision-making in relation to the identification and management of patients with possible oral cancer, based, in part on the previously published “RULE” for oral cancer diagnosis (lesions that are Red or white; Ulcerated; Lump; Extending >3 weeks) (6). This tool is based on the grading of general clinical observations along the green to red spectrum by the examining dental professional that should allow a degree of confidence within an office or clinic setting for identification of signs and habits related to oral cancer causation and evolution ( Table 1).

**Table 1:**
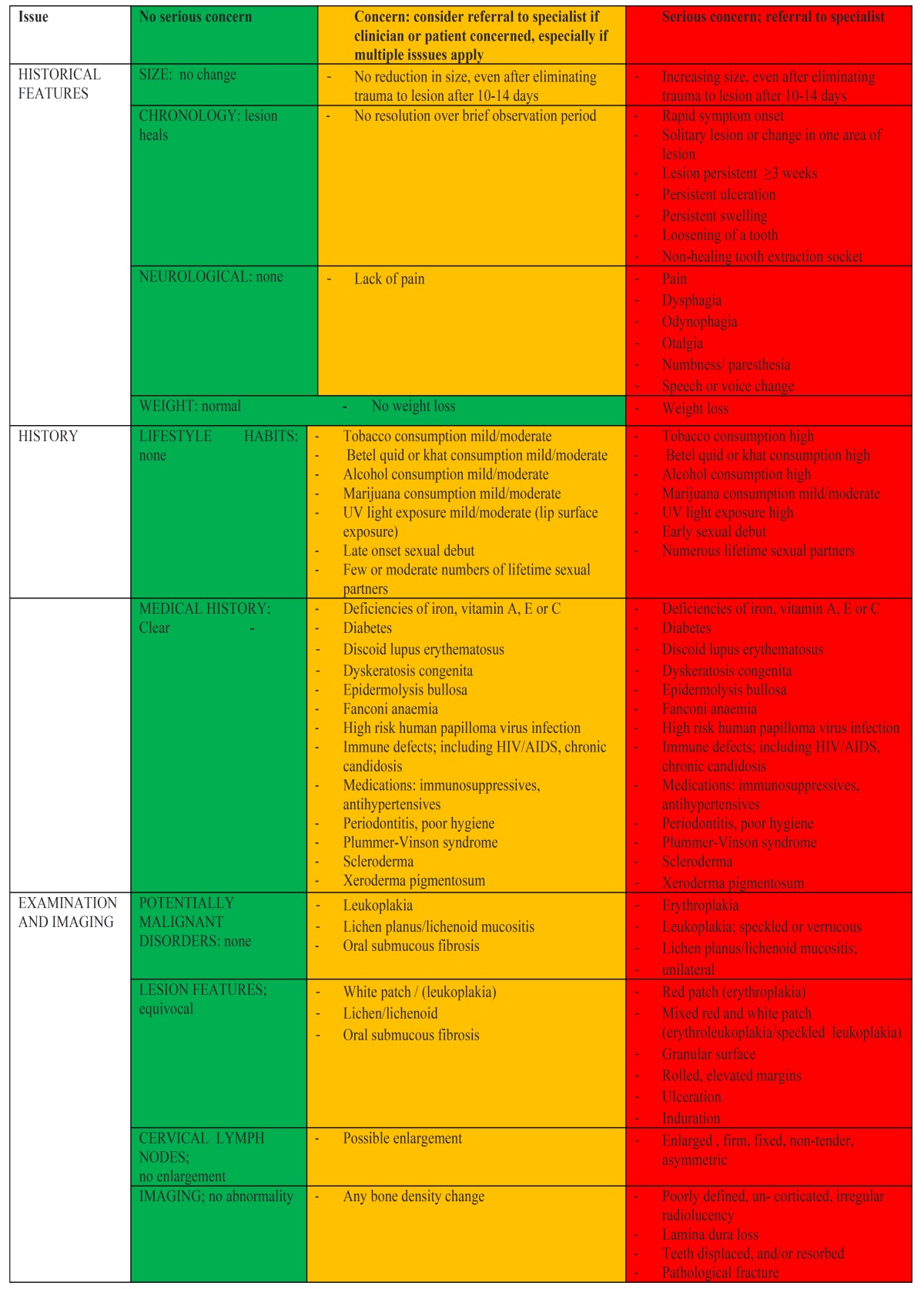
Oral mucosal lesions symptoms and signs.

The straightforward tool or scheme we have developed, addresses essential considerations in measurement, recognition and prevention of oral cancer development and progression and the role played by the dental professional in recognition and appreciation of the historic and clinical factors in an all too common disease. Deviations from recognized clinical norms therefore, with either referral or direct action on the part of clinicians, thus can provide the best chance for reducing the frequency of errors and corresponding improvement of treatment outcomes. Finally, this approach could serve as a working template for all initial and future oral examinations, even in the absence of prior clinical indicators of mucosal abnormalities.
